# Antiobesity effect of healthy food crops and functional foods: A systematic review of their mechanisms

**DOI:** 10.1002/fsn3.3856

**Published:** 2023-11-20

**Authors:** Beatrice Mofoluwaso Oladimeji, Oluwafemi Ayodeji Adebo

**Affiliations:** ^1^ Food Innovation Research Group, Department of Biotechnology & Food Technology, Faculty of Science University of Johannesburg Johannesburg South Africa

**Keywords:** food crops, functional foods, mechanisms of action, obesity management

## Abstract

Diet is a modifiable risk factor in the prevention and management of obesity, and various foods have the potential to aid in obesity management by modulating different pathways involved in the disease's pathology. We performed a systematic review of literature, using CINAHL, PubMed, and Google Scholar, focusing on the antiobesity potential of foods crops and functional food products, and their mechanisms of action and clinical evidence. Sixty‐four articles were identified, of which 41 investigated food crops, while 23 investigated functional products. Food crops, such as cereals, vegetables, fruits, mushrooms, seaweeds, legumes, herbs, spices, and cocoa seeds, have antiobesity effects through mechanisms such as altering the metabolism of glucolipids by inhibiting enzymes like α‐amylase and α‐glucosidase, stimulating the bioenergetics of thermogenic fat, modulating gut microbiota, and inhibiting lipogenesis and storage. In addition, developed functional teas, beverages, and yoghurt have antiobesity effects through similar or different mechanisms, such as enhancing energy expenditure and satiety, suppressing adipogenesis and lipolysis, improving glucose and lipid metabolism, and altering hormonal secretion. This review reemphasized the significance of food in the control of obesity, and highlights the distinct methods these explored foods exert their antiobesity effects. In conclusion, foods are safe and effective means of combating obesity without the side effects of conventional drugs, which can help inform dietary choices, assist professionals in providing more accurate advice, and also lead to better understanding of food and its effect on overall health of the public. This approach will eradicate global diseases, especially if more underutilized and indigenous food crops are extensively researched.

## Graphical abstract

Obesity is of global concern, and the exploitation of several food crops has shown great prospects for management. The food component modified gut microbiota showing antiobesity effects. Thermogenesis and lipolysis are other common mechanisms of action, while enzymes involved in glucolipid metabolism are the major pathway explored.

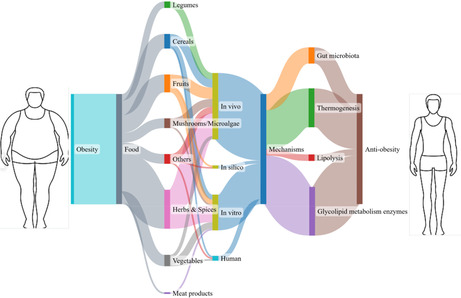

## 1 INTRODUCTION

There is an alarming health problem when an individual's body weight in kilograms is measured against the height in meters squared and the resulting body mass index (BMI) is high (Chooi et al., 2019). This means that there is an abnormal or excessive accumulation of fat in the body, a condition commonly referred to as obesity. Obesity (overweight) has become a growing health burden in modern society, with exponentially increasing rates projected to exceed 1 billion by 2030 (World Obesity Federation, 2022). The public frequently perceives obesity as the result of a lack of self‐control, leading to poor dietary choices and physical inactivity. Research has however described obesity as a complicated multifactorial chronic medical condition caused by the interaction of genetic, metabolic, environmental, and behavioral factors (Smith & Smith, 2016; Upadhyay et al., 2018). Obesity harms nearly all physiological functions of the body, increasing the risk of developing multiple disease conditions such as diabetes mellitus, cardiovascular disease, several types of cancer, some forms of musculoskeletal disorders, decreased fertility in women, and poor mental health, all of which have a negative impact on quality of life, productivity, and healthcare costs (Chooi et al., 2019).

Obesity might start off during childhood and can significantly increase through adolescence (Craigie et al., 2011), resulting in several comorbidities and high mortality during adulthood (Blüher & Schwarz, 2014). Approximately 80% of obese adolescents continue to be obese into adulthood (Simmonds et al., 2016). Along with other contributing environmental and behavioral factors, genetic susceptibility has been identified as the leading cause of obesity in both children/adolescents and adults (Sahoo et al., 2015). Diets also play a significant role in obesity as reports have linked obesity to unhealthy dietary patterns with diets high in fats, and sugars (high‐energy diet) being implicated in rapid weight gain (Liberali et al., 2020; Mustafa et al., 2021).

There is a growing obesity epidemic with related health issues throughout Africa as well. The shift in the population's dietary habits from the traditional African cuisine to a western diet and a growing decrease in physical activity have been connected to the rising (from 4.5% to 32.5%) prevalence across the continent, with higher frequency observed in metropolitan areas (Azeez, 2022). However, a return to the traditional diet reduced obesity among rural residents (Aborode et al., 2023).

One of the recommended healthy ways of preventing or managing obesity is through caloric and micronutrient intake control. Dietary patterns that properly balance fats, proteins, carbohydrates, and micronutrient composition can result in weight loss, and can thus be used to manage obesity (Shatwan & Almoraie, 2022). Adding functional foods to the diet can also be a strategy for treating obesity. Foods and dietary ingredients that offer health advantages over and above basic nourishment are known as functional foods (Sunkara & Verghese, 2014). They include biologically active ingredients that have positive physiological effects or health advantages. Foods that include significant components and chemicals that have antiobesity properties may be useful in the management of obesity (Sunkara & Verghese, 2014). Foods high in dietary fiber, glutamine, soy peptides, or calcium, for instance, can supplement a diet to promote antiobesity effects by regulating energy metabolism, enhancing energy expenditure, decreasing intestinal cholesterol absorption, and modifying gut microbiota and fat oxidation (Sunkara & Verghese, 2014). For the purpose of this review, however, functional foods are defined as foods or beverages that have been specially prepared or enhanced with the intention of managing obesity.

Despite knowledge of obesity and the role that diet plays in the etiology of the disease, obesity remains a global problem, with many more people becoming obese each year with a notable global increase in the previous 50 years (Lin & Li, 2021). The underlying conflict between environment and microenvironmental factors, genetics and epigenetics, and nature versus nurture has been connected to this rising occurrence. Current epigenetic research has also demonstrated the importance of epigenetic variables in metabolism control, obesity risk, and associated comorbidities (Lin & Li, 2021). Furthermore, the knowledge about antiobesity food crops and products and the availability and accessibility of these food crops have been underrated. The goal of this review is to assess recent research on food crops with antiobesity potential and functional food products developed for the management of the disease. As scientific research and reviews exist on this topic of research, yet obesity is increasing in the world especially in Africa where several crops with antiobesity potential exists. This review will hearten the exploration of several underutilized and indigenous food crops and the production and development of functional foods from these crops.

## 2 METHODOLOGY

### 2.1 Literature search

A review of the evidence was conducted using the guidelines of the Preferred Reporting Items for Systematic Reviews and Meta‐analysis (PRISMA; Moher et al., 2015) checklist to assess food crops with antiobesity potential and functional foods developed for the management of obesity. The search was conducted on January 20 and 21, 2023 and all relevant studies were obtained in summary form for screening.

### 2.2 Search process

Google Scholar (GS), a commonly web‐based academic and gray literature search engine, has been reported to be powerful tool during systematic review case studies (Haddaway et al., 2015) if used in addition to other multiple databases. A systematic literature search was conducted in Google Scholar, PubMed (MEDLINE – Preclinical research), and CINAHL (EBSCO – Cumulative Index to Nursing and Allied Health Literature) search engines and databases for studies published in the last 10 years that discussed food crops and functional food products used for obesity management. Appropriate search terms were used, and the search was carried out using the index words “food crops” + “obesity management” or “functional foods” + “obesity management.” Some of the retrieved articles' reference lists were also checked for additional relevant publications. To narrow down the search results, criteria such as “full text only,” “original article,” and “peer‐reviewed journals” were used.

The studies were chosen by evaluating the titles and abstracts of the retrieved articles, and studies whose full texts were unavailable were discarded. Abstracts of retrieved articles that met the inclusion criteria were kept, while those that did not were discarded. The full texts of the potentially eligible articles were evaluated further to determine their eligibility. Articles were considered for inclusion if they met the following criteria: (1) experimentation with edible food crops in the management of obesity, either using animal models or human studies using randomized controlled trials or clinical studies, (2) experiments involving developed functional foods, teas, drinks, or plant materials used primarily for drinks or teas used in the management of obesity, either using animal models or human studies in a randomized controlled trial or clinical studies, and (3) experiment with isolated bioactive compounds from some food crops used in the treatment of obesity. Articles are excluded if: (1) they are experimental studies involving medicinal plants rather than food crops, (2) they are experiments with nonfood parts of food crops, (3) they are review articles, and (4) they are preprints or articles that have not been peer reviewed.

### 2.3 Relevant publications selected

Sixty‐four articles were chosen and examined in light of the study's goal. Eligible articles contained information on food crops or functional foods for obesity management. The data were extracted by organizing the various articles by topic, with those dealing with food crops organized separately from those dealing with functional foods. Different food classes were determined from the retrieved data, together with their mechanisms of action and research methodologies. The publications contained information on the author's name, year of publication, methods, the country in which the study was conducted, the study design, and the types of subjects used.

## 3 RESULTS

**FIGURE 1:**
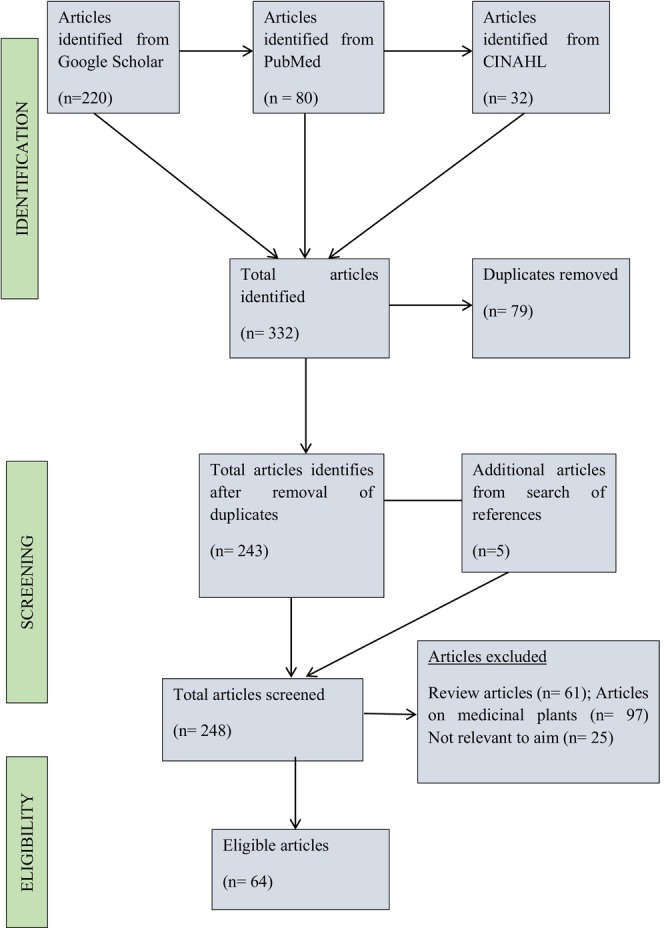
PRISMA flow diagram of the final search.

After conducting a thorough literature search, this yielded 332 publications. Excluding duplicates, review articles, research on medicinal plants, and other items that were not related to the study's aim, 64 articles were included in the study (Figure 1). Forty‐one of the 64 articles focused particularly on antiobesity food crops. This research looked at the antiobesity potential of food crops as extracts, fermented products, food crops combined with other food crops, bioactive fractions, or isolated bioactive components. However, 23 of the publications examined functional foods that have the potential to be employed in the therapy of obesity. The functional foods include drinks or teas, snacks, sausages, food additives, legume paste, and jelly, many of which were developed or have traditionally been used to treat obesity and other metabolic diseases.

### 3.1 Food crops with potential benefits in the management of obesity

Forty‐one publications investigated food crops used in the treatment of obesity. The majority of the publications utilized are from the Asian continent, with China contributing the most with 12 publications. South Korea had seven publications; Italy had three, while Malaysia and Nigeria each had two. Japan, Taiwan, India, Cameroon, Iran, the United States, and Jordan each had one publication, while partnerships among South Korea/Vietnam, Pakistan/Belgium/Malaysia, Malaysia/Netherlands, Peru/Chile/USA, Iran/Turkey, Pakistan/Malaysia, Germany/Spain, and Mexico/Spain all had one publication each.

Each of these separate studies used a different technique of inquiry, with 23 of the 41 investigations being purely animal trials with rodents (rats/mice). There were nine in vitro trials; seven studies carried out both in vitro and in vivo (animal tests), and one study was conducted in vitro/in silico and in vivo. Furthermore, how the crops were used varied in the research. As revealed in Table 1, 12 studies used extracts (ethanolic, aqueous, methanolic, or lipophilic) of the crop, six studies used the whole crop, seven other studies used the bioactive fractions (polyphenols‐rich, fucoxanthin‐rich, glabridin‐rich fractions) of the plants. Four studies used extracts of fermented plants, two studies isolated bioactive components (Nguyen et al., 2016; Wang et al., 2020), polysaccharides (Huang et al., 2014; Yang et al., 2020), and essential oils (Bahadori et al., 2017; Zhang et al., 2019) from the food crop of interest. Enzyme‐treated extract (Cho et al., 2020), seed proteins of a plant (Coronado‐Cáceres et al., 2020), juice (Wu et al., 2013), both extract and isolated bioactive component (Kianbakht & Hashem Dabaghian, 2015; Lee et al., 2018), and whole plant combined with other food crops (Okouchi et al., 2019) were also explored.

**TABLE 1:** Summary of the examined food crops with potential benefits in the management of obesity.

Food source	Methods of evaluation	Findings	Conclusion	References
Cereals				
Whole barley (*Hordeum* L. var. *nudum* Hook. f.)	In vivo	Whole barley prevents obesity via inhibition of cholesterol synthesis independent of gut microbiota and counteracts gut dysbiosis in obese mice.	Whole barley prevents obesity via both gut microbiota‐dependent and gut microbiota‐independent mechanisms.	Gong et al. (2019)
Barley extracts (*Hordeum vulgare* L.) Yangsi‐3 variety	In vitro/in vivo	In vitro, fermented barley extracts reduced adipogenesis while stimulating browning in 3T3‐L1 preadipocytes. In vivo it reduced body weight by increasing brown adipose tissue (BAT) mass. This is accomplished by activating the uncoupling protein 1 (UCP1)‐dependent pathway to decrease obesity through thermogenesis and epididymis adipose tissue (EAT) browning.	Barley extracts fermented with *Lactobacillus plantarum* dy‐1 may be useful in the management of obesity via UCP1‐dependent mechanisms.	Xiao et al. (2019)
Barley extracts (Yangsi‐3)	In vivo	*Lactobacillus plantarum* dy‐1 fermented barley extracts reduced body weight, suppressed visceral lipid accumulation and improved glucolipid metabolism in obese rats by activating thermogenic fat bioenergetics and mitochondria biosynthesis.	Consumption of fermented barley may be beneficial in inhibiting diet‐induced obesity, without suppressing energy intake by enhancing the expression of UCP1 level.	Gu et al. (2021)
Corn (*Zea mays* L.)	In vitro	Peruvian corn has inhibitory activities against some glucolipid metabolism enzymes such as lipase, α‐amylase, and α‐glucosidase.	Peruvian corn may have antiobesity potential, linked to their anthocyanins, phenolics, and antioxidant bioactivities.	Ranilla et al. (2019)
Sorghum bran (extracts of PI570481; SC84/PI534144; Sumac; and white sorghum varieties)	In vitro	In differentiated 3T3‐L1 cells, sorghum bran extracts inhibited intracellular lipid accumulation and the production of adipogenic and lipogenic proteins. The extracts also inhibited the generation of reactive oxygen species (ROS) and MAPK signaling pathways, as well as insulin signaling and glucose absorption.	Sorghum bran may have antiobesity properties due to its antioxidant activity and regulation of glucolipid metabolism.	Lee et al. (2022)
Rice (*Oryza sativa* MR220 and MR219)	In vivo	Germinated brown rice lowered adiposity by decreasing white adipose tissue bulk, adipocyte size, and leptin levels, as fat was expelled in the feces of the obese rats.	Germinated brown rice promoted satiety and reduced body weight while improving lipid profiles. Thus can be utilized as antiobesity food because of the synergistic effects of its dietary fiber and other bioactive compounds.	Lim et al. (2016)
Flaxseed	In vivo	Flaxseed polysaccharides lowered fasting glucose, total triglyceride, and total cholesterol levels in the blood. Its build‐up in adipose tissues influenced the gut microbiota by raising the proportions of beneficial *Akkermansia* and Bifidobacterium and lowering the proportions of obesity‐causing Oscillospira and Odoribacteraceae.	By altering gut microbiota and glucolipid metabolism, flaxseed polysaccharides have the potential to be beneficial in the therapy of metabolic syndromes such as obesity	Yang et al. (2020)
Fruits				
Pomegranate, blood orange, and mulberry juice	In vitro	Fruits with high anthocyanin content can strongly inhibit the activity of pancreatic lipase, thereby inhibiting fat accumulation and weight gain.	The total anthocyanin content of these fruit extracts inhibited pancreatic lipase activity, thus possessing antiobesity effect.	Fabroni et al. (2016)
Plantain (*Musa* spp. AAB)	In vivo	Unripe plantain flour lowers insulin levels, positively regulates the composition of the gut microbiota, preventing fat accumulation and weight gain. It also changes the expression of genes involved in glucolipid metabolism, by activating peroxisome proliferator receptor and protein kinase signaling pathway genes.	The resistant starch and dietary fiber of the unripe plantain flour may have effected lipid metabolism, causing a reduction in the serum total cholesterol and fat accretion.	Fu et al. (2022)
Mulberry leaf (*Morus alba* L.)	In vivo/in vitro	Mulberry leaf consumption reduces body weight gain and lipid accumulation in the liver of mice fed a high‐fat diet by suppressing the expression of genes associated with lipogenesis and inhibiting the differentiation of 3T3‐L1 preadipocytes.	Mulberry leaf could be effective in the management of obesity, due to its inhibition of lipogenesis through the regulation of fatty acid synthetase.	Chang et al. (2016)
Soursop (*Annona muricata*)	In vivo	Administration of soursop in high‐fat diet‐induced obese rats resulted in a downregulation of the fat mass and obesity‐associated protein (FTO), and an upregulation of the STAT‐3 gene.	Soursop has beneficial antiobesity potential and its mechanism of action leads to reduction of food intake due to the effect of annonaine and annonioside.	Elekofehinti et al. (2020)
Cocoa (*Theobroma cacao* L.)	In vitro/in silico and in vivo	Cocoa protein hydrolysates inhibited pancreatic lipase similarly to Orlistat (a typical antiobesity medicine), but with less hepatotoxicity in vitro. It also triggered increased fat excretion in the feces of obese rats on a high‐fat diet.	Cocoa protein hydrolysates may have an antiobesity impact as peptides that inhibits obesity‐related molecular targets are released during gastrointestinal hydrolysis.	Coronado‐Cáceres et al. (2020)
Cocoa	In vivo	Cocoa polyphenols lower lipids in the liver, reduce body weight, and prevent visceral fat accumulation in rats induced with obesity using a high‐fat diet by upregulating genes associated with lipid catabolism and downregulating genes associated with lipid synthesis.	Cocoa may be useful in the treatment of diet‐induced obesity, for it polyphenolic compounds.	Ali et al. (2015)
Blueberry and mulberry juices	In vivo	In C57BL/6 mice fed a high‐fat diet (HFD), blueberry and mulberry juice prevented body weight growth, lowered blood cholesterol, reduced insulin resistance, attenuated lipid build‐up, and decreased leptin secretin.	Blueberry and mulberry juice may help fight obesity, as their anthocyanin and total phenolic contents are effected on the loss of body weight.	Wu et al. (2013)
Blueberry	In vivo	The administration of blueberry polyphenols to obese C57BL/6J mice restored their weight while also modifying lipid metabolism via the regulation of gut microbiota composition by boosting helpful bacteria and decreasing dangerous bacteria.	Blueberry may help fight obesity, as the polyphenol extracts act as a prebiotic, and so modifying gut bacteria.	Jiao et al. (2019)
Others				
Mushroom (*Pleurotus eryngii* var*. ferulae*)	In vitro	Mushroom inhibits key adipogenic factors thus suppressing adipogenesis in 3T3‐L1 cells	Mushrooms are potential antiobesity food, as this variety reduced the accumulation of lipid, by controlling lipid and glucose metabolism.	Kang et al. (2016)
Mushroom (*Inonotus obliquus*)	In vivo	Fermented *Chaga‐cheonggukjang* mushroom improves lipid profile, reduces body weight, and reduces epididymal fat pad weight gain in obese mice fed a high‐fat diet.	Mushroom fermented with *Lactobacillus acidophilus* has an antiobesity effect, which may be associated to its isoflavones.	Na et al. (2019)
Mushroom (*Pleurotus tuber‐regium*)	In vivo	Polysaccharides extracted from the edible mushroom were found to lower weight growth and epididymal body fat in rats induced with high‐fat diet obesity. It also enhances glucolipid metabolism by increasing PPAR‐α mRNA and protein levels in the liver.	Mushroom polysaccharides may be useful in the treatment of obesity by stabilizing fatty acid components in the liver and plasma of obese diabetic rats.	Huang et al. (2014)
Microalgae (*Euglena gracilis*)	In vivo	Co‐consuming microalgae with some vegetables, significantly reduced the accumulation of visceral fat in obese mice via the suppression of genes involved in fatty acid synthesis and the upregulation of genes involved in adipocyte lipolysis.	Microalgae has an antiobesity effect via the modulation of gut microbiota composition.	Okouchi et al. (2019)
Microalgae (extract of *Phaeodactylum tricornutum*)	In vivo	In diet‐induced C57BL/6J obese mice, ethanolic extract of *P. tricornutum* decreased body weight gain and the weight of white adipose tissues (WAT) while also reducing the expression of Mest (a marker of fat tissue expandability) in WAT depots. Also, the expression of genes involved in lipid intake was reduced while increasing the expression of genes involved in fatty acid oxidation and thermogenesis.	Microalgae have the capacity to reduce high‐fat diet‐induced obesity in vivo, connecting to this effect to stimulation of oxidative metabolism in BAT and WAT.	Gille et al. (2019)
Brown algae (seaweeds)	In vivo	Laminarin (a major polysaccharide in edible seaweeds) reduced weight gain in mice by modulating the gut microbiota in favor of energy metabolism via an increase in beneficial microbes (Bacteroides) and a decrease in nonbeneficial microbes (Firmicutes).	Laminarin has high potential as a prebiotics, as it increases bacteria that digest dietary polysaccharides and decreased potentially pathogenic bacteria. Hence could potentially be used to prevent or manage obesity.	Nguyen et al. (2016)
Legumes				
Soybeans	In vivo	Soybean extract has slimming and serum lipid regulatory abilities, which is affected by its cooking procedures.	Unhulled soybean extract and unhulled boiled soybean have antiobesity potential due to the ability of their high phenolic content, flavonoid and fiber content in reducing total serum cholesterol.	Woumbo et al. (2017)
Beans (*Phaseolus vulgaris* L.)	In vivo	Beans extract reduced body weight gain and improved blood lipid levels in obese rats fed a high‐fat diet. This is accomplished by lowering the relative abundances of Firmicutes and Proteobacteria while raising the relative abundances of Bacteroidetes and *Akkermansia* in the gut microbiota.	White common bean extract may have antiobesity properties due to its high α‐amylase inhibitory effect.	Shi et al. (2020)
Horse gram (*Macrotyloma uniflorum*)	In vivo	*Macrotyloma uniflorum* reduced body weight and improved the lipid profile of rats induced with metabolic syndrome using a high‐fat diet. It also influenced the expression of genes involved in adipogenesis and lipid metabolism.	Polyphenols and polyunsaturated fatty acid in the legume may be responsible for its potential use in the treatment of metabolic disorders such as obesity, diabetes, and cardiovascular disease.	Malarvizhi et al. (2021)
Herbs and spices				
Ginger (*Zingiber officinale* Rosco)	In vivo	Ginger administration prevents body weight gain, modulates energy metabolism, and induces the browning of white adipose tissues in obese mice fed a high‐fat diet.	Ginger has the potential to treat obesity and other related disorders, since its bioactive constituents can alter some lipid metabolism activities.	Wang et al. (2019)
Cinnamon (*trans*‐cinnamic acid)	In vitro/ In vivo	*Trans*‐cinnamic acid reduced lipid accumulation in HepG2 cells exposed to oleic acid while also decreasing body weight growth, liver and adipose tissue weight, and improved hepatic steatosis and adipose hypertrophy in mice fed a high‐fat diet. It also improved the lipid profile of mice on a high‐fat diet.	Bioactive components in cinnamon improves lipid metabolism, thus have an important role in the prevention and treatment of diet‐induced obesity.	Wang et al. (2020)
Garlic (*Allium sativum*) oil	In vivo	Garlic oil treatment, either orally or by Shenque point injection, dramatically lowered body weight while improving lipid profile in rats fed a high‐fat diet, albeit Shenque point administration was shown to be more effective.	Garlic oil has the potential to be employed in the treatment of obesity.	Zhang et al. (2019)
Turmeric (*Curcuma longa* L.)	In vivo	Fermented turmeric repressed body weight growth, decreased white adipose tissue weight, and improved lipid profile in high‐fat diet‐induced obese mice. This is related to the suppression of adipocyte differentiation and lipogenesis, which is accompanied by a decrease in the expression of genes involved with fatty acid production and adipogenesis while increasing the expression of genes associated with fatty acid oxidation and lipolysis.	Fermented turmeric may be useful in the prevention of obesity by decreasing adipogenesis and increasing lipolysis.	Kim et al. (2016)
*Prangos gaubae* oil	In vitro	The essential oil of *P. gaubae*, a spice used in the Middle East, inhibited α‐amylase, α‐glucosidase, and lipase, altering glucolipid metabolism.	The essential oil of *P. gaubae* possess antioxidant properties, making them useful in the treatment of a variety of ailments, including obesity, and cardiovascular disease.	Bahadori et al. (2017)
Edible flower (*Capparis sicula* and *Borago officinalis*)	In vitro	*Capparis sicula* and *Borago officinalis* are edible Mediterranean plants that inhibit pancreatic lipase and α‐amylase, respectively, assisting in the regulation of glucolipid metabolism.	These plants may be useful in the treatment of obesity	Marrelli et al. (2014)
Basil (*Ocimum basilicum*)	In vitro	The extracts of basil leaves and flowers are high in antioxidants, estragole and linalool, which can bind strongly to pancreatic lipase and α‐amylase, inhibiting their activities.	High antioxidant content of basil makes them useful in the treatment of several cardiovascular diseases, including diabetes and obesity.	Noor et al. (2019)
*Cosmos caudatus kunth* leaf extract	In vivo	Ethanolic extract of *Cosmos caudatus kunth* leaf decreased weight growth and enhanced levels of obesity indicators such as lipid profile, insulin, ghrelin, and adiponectin in rats fed a high‐fat diet, while also improving fecal fat excretion.	*Cosmos caudatus* ethanolic extract may be useful as an antiobesity agent due to its ability to suppresses pancreatic lipase activity.	Rahman et al. (2017)
*Taraxacum officinale* extract	In vitro/in vivo	Hydroethanolic extract of *Taraxacum officinale* inhibited pancreatic lipase in vitro, with metabolites such as myricetin, isomangeferin, and kaempferol contributing significantly to the molecular binding energies. In vivo studies in obese mice revealed a positive impact on lipid profile and obesity biomarkers.	*Taraxacum officinale* may have therapeutic use in the treatment of obesity, because of metabolites, and positive impact on lipid metabolism.	Aabideen et al. (2020)
Saffron extract	In vivo	In obese Wistar rats, saffron extract and its bioactive component, crocin, lowered body weight, food intake, and leptin levels. It also decreased body fat while boosting insulin sensitivity.	The bioactive component of saffron may be useful as antiobesity agents.	Kianbakht and Hashem Dabaghian (2015)
Sage	In vivo	Sage inhibits pancreatic triacylglycerol lipase, reversing HFD‐induced postprandial hypertriglyceridemia, and has an antilipolytic action, decreasing body weight gain in high‐fat diet‐induced rats.	Dietary sage may help to reduce weight gain caused by a high‐fat diet.	Arabiyat et al. (2016)
Licorice (*Glycyrrhiza uralensis*)	In vitro/in vivo	In 3T3‐L1 adipocytes, extracts of *G. uralensis*‐induced expression of uncoupling protein‐1 (UCP‐1), a fat browning marker. Intraperitonial administration of *G. uralensis* extract reduced body weight gain and inguinal fat pad weight while also lowering serum glucose and cholesterol levels and blocking insulin resistance. They also promote brown fat.	*Glycyrrhiza uralensis* and licochalcone A have potential benefits in decreasing obesity and restoring metabolic balance via the development of the brown fat phenotype.	Lee et al. (2018)
Licorice component (Glabridin)	In vitro/in vivo	Glabridin suppressed adipogenesis in 3T3‐L1 cells. It also suppresses adipogenesis, reduced weight gain in the high‐fat diet in rats while also reducing hypertrophy of white adipose tissue and fat cell size, inhibited high‐fat diet‐induced hepatic steatosis.	The bioactive component of licorice (glabridin) might be a potent antiobesity agent.	Ahn et al. (2013)
Vegetables				
Wild carrot (*Daucus carota* L.)	In vitro	*Daucus carota* demonstrated antiobesity activity via pancreatic lipase inhibition, with the potential to reduce or prevent weight gain.	*Daucus carota* possesses the antiobesity potential, significantly for its bioactive constituents.	Marrelli et al. (2020)
*Allium* genus	In vitro	Wild vegetables (*Allium tuberosum* Rottl., *Allium senescens* L., *Allium thunbergii* G. Don., and *Allium sacculiferum* Maxim.) showed antiadipogenic and antioxidant activities, with *A. tuberosum* and *A sacculiferum* showing superior activities due to the presence of caffeic acid in their extracts.	These wild vegetables may have antioxidant and antiobesity properties; however, their efficiency varies due to differences in phenolic content.	Lee et al. (2021)
Bitter leaf (*Vernonia amygdalina*)	In vivo	The administration of aqueous and methanolic extracts of bitter leaves resulted in weight loss in obese rats while having no adverse impact on internal organs and improved several metabolic indicators of obesity such as lipid profile.	*Vernonia amygdalina* may have antiobesity properties, since its phytochemicals increased metabolic efficiency.	Egedigwe et al. (2016)
Celery extract	In vitro	Celery extract treated with enzymes shown in vitro antiadipogenic activities by inhibiting lipid accumulation in adipocytes, whereas treatment decreased high‐fat diet‐induced body weight gain, epididymal fats, and liver weights in rats.	The use of enzyme‐treated celery extract may aid in the prevention and control of obesity.	Cho et al. (2020)

Food crops with antiobesity potential discovered in this study come from a variety of dietary groups. Six studies focused on cereals, three each on edible mushrooms and edible seaweeds, while fruits were the focus of six other studies. Other focused on flour (Fu et al., 2022), three studies on legumes, thirteen studies concentrated on herbs and spices, two studies were on cocoa seeds, and three others on vegetables.

### 3.2 Functional foods with potential benefits in the management of obesity

Twenty‐four publications considered functional foods to have antiobesity potential. Some of these foods are plants that have been traditionally used for this purpose or whose usefulness in this respect is only becoming known, while others are foods that have been intentionally designed to incorporate these specific health advantages. The majority of the publications utilized are from Asia, with China having seven publications, South Korea having three publications, and Japan, Malaysia, and Iran having one publication each. Iran/Italy/Turkey, Egypt/Saudi Arabia, and Iran/Canada are all examples of partnerships between one or more countries. Other nations that contributed include Spain, Cameroon, Australia, the United States, and Egypt, each of which produced one publication. Other partnerships that produced one publication each include Brazil/United Kingdom, Mexico/Spain, and Mexico/USA.

**FIGURE 2:**
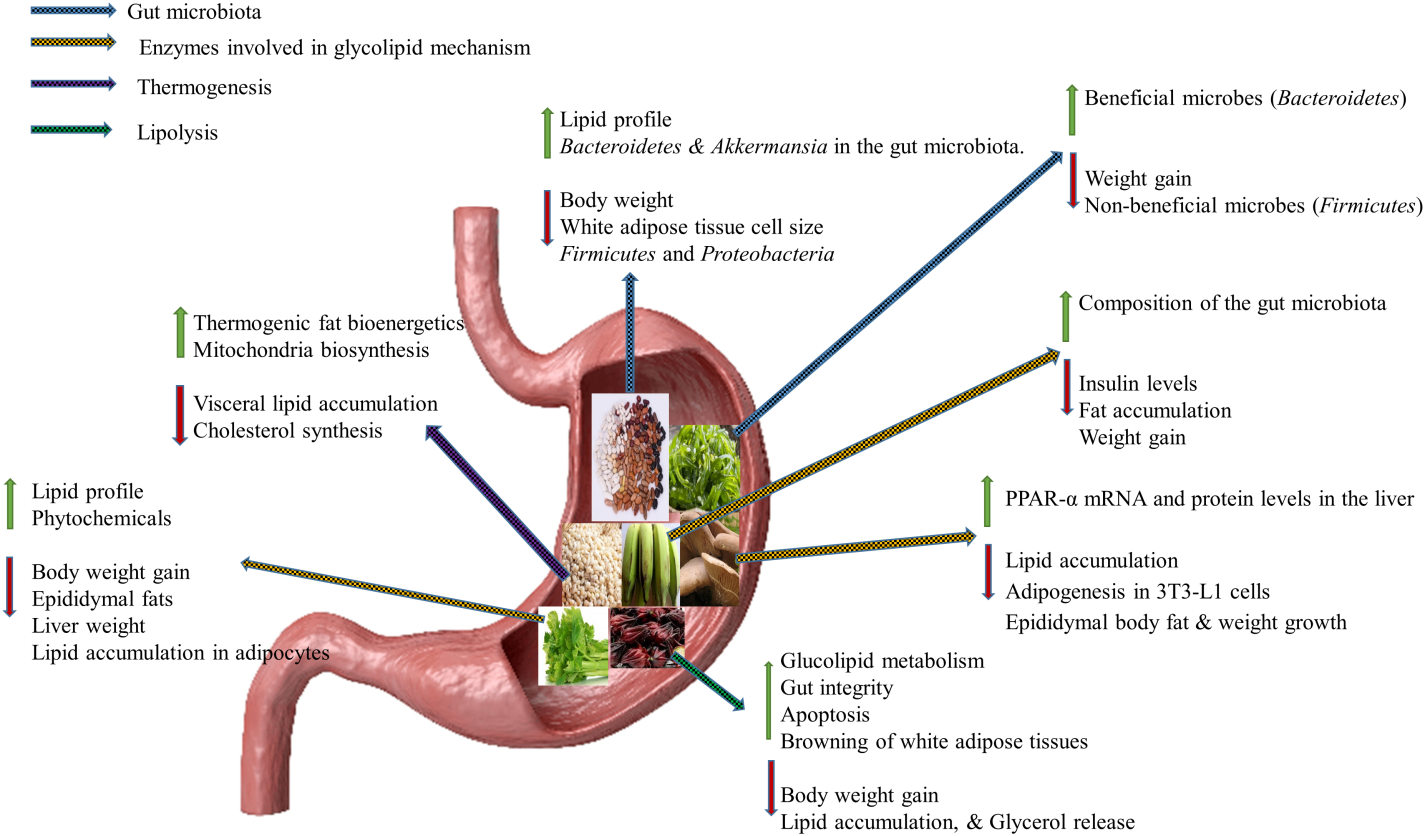
A summarized mechanism of action by which food crops and functional foods contributes to the prevention and treatment of obesity.

Many research employed various methodologies to test the antiobesity effects of the functional meals/products created. Thirteen of the 23 studies were animal trials employing rodents (rats and mice), whereas one study included *Caenorhabditis elegans* (a nematode; Lin et al., 2021). Four other studies were in vitro experiments, and three were randomized controlled trials in humans. One research incorporated in vitro and in silico tests (Uddin et al., 2022), and another in vitro and animal trial (Lee et al., 2023). The functional foods were also presented in various forms including beverages, teas, cereal meals, sausage (Qi & Zhou, 2013), vegetable juice (Lee et al., 2023), soybean paste (Nam et al., 2015), jelly (Peng et al., 2022), yoghurt (Mohammadi‐Sartang et al., 2018), food additive (Li et al., 2019), and honey propolis (Uddin et al., 2022), as shown in Table 2. The summary of some the reported mechanisms of action are described in Figure 2.

**TABLE 2:** Summary of the evaluated functional foods with potential benefits in the management of obesity.

Food product	Method	Findings	Conclusion	References
Cereals				
Cereals (rice) husk and ban meal	Human	Obese people were given rice husk powder and rice bran together with a calorie limited diet, which lowered their BMI, waist circumference, and serum levels of inflammatory markers (high sensitivity C‐reactive protein [hs‐CRP] and interleukin‐6 [IL‐6]).	Rice bran and rice husk powder had a favorable effect on lowering overweight/obesity and inflammation, when paired with an energy‐limited diet because of its dietary fiber content.	Edrisi et al. (2018)
Extruded puffs	In vivo	Extruded puff from a mix of cereals, tubers, and legumes (millet, maize, oat, soybean, and purple potato) fed to rats on a high‐fat diet, reduced their body weight gain and fat accumulation while improving glucolipid metabolism through the downregulation of hepatic lipogenic genes. It also boosts the production of SCFAs, increases the variety of gut microbiota, and increases the relative abundance of *Lactobacillus* and *Bifidobacterium*, all of which may contribute to antiobesity effects.	Coarse cereal rich in dietary fiber, resistance starch, β‐glucan, and polyphenols enhances gut microbiota, which might be functional to prevent HFD‐induced obesity.	Ji et al. (2021)
Herbs and spices				
Roselle calyx extract	In vivo	*Hibiscus sabdariffa* extracts lowered weight gain in mice fed a high‐fat diet (HFD) and enhanced glucolipid metabolism. It also improved gut integrity by boosting the expression of mucins and proteins involved in mucosal barrier maintenance.	*Hibiscus sabdariffa* might be used to treat obesity, as it had a prebiotic impact, reducing the effects of the high‐fat diet on the gut flora.	Diez‐Echave et al. (2020)
Roselle calyx extracts	In vitro	Extracts of *H. sabdariffa* reduced the activities of α‐glucosidase, α‐amylase, and pancreatic lipase. Thus, in 3T3‐L1 cells, lipid accumulation is reduced, apoptosis is induced, lipolysis is increased, and glycerol release is increased.	Phytochemical and antioxidant content may be responsible for the usefulness of *H. sabdariffa* in the treatment of obesity and other glucolipid metabolic problems.	Mutai et al. (2015)
Roselle calyx powder	In vivo	In rats fed a high‐fat diet, a combination of roselle calyx powder and dietary fiber derived from its byproducts decreased body weight gain, adipocyte hypertrophy, insulin resistance, and hepatic steatosis.	Roselle calyx and its byproduct might be employed as a functional food element in the treatment of obesity.	Amaya‐Cruz et al. (2019)
Roselle calyx	In vivo	*Hibiscus sabdariffa* lowered body weight increase and abdominal weight in obese rats fed a high‐fat diet.	Malaysian *H. sabdariffa* may be used in the treatment of obesity.	Omar et al. (2018)
Teas	In vitro	The inclusion of immobilized tannase in teas, black, green, white, or mate teas, significantly increased their ability to inhibit lipid build‐up in adipocytes. It also increased their ability to inhibit the enzymes α‐amylase, α‐glycosidase, and lipase.	Polyphenol content may boost the advantages of tea as antiobesity food.	Roberto et al. (2016)
Teas	In vivo	The fermented mixed tea considerably reduced rats' body weight and adipose tissue weight while also lowering the blood and hepatic triglycerides by inhibiting the activities of hepatic lipogenic enzymes.	Blended tea may be effective in the treatment of hypertriglyceridemia and obesity.	Tamaru et al. (2013)
Teas	In vivo	In obese rats fed a high‐fat diet, Fuzhuan brick tea, a microbially fermented tea, reduced body weight increase, adipose tissue buildup, and improved lipid profile. It also reduced the expression of enzymes and factors involved in lipogenic metabolism while increasing the expression of enzymes involved in energy expenditure and lipodieresis.	Fuzhuan brick tea possesses polyphenols and flavononoids, implying their antiobesity and hypolipidemic potentials.	Li et al. (2013)
Teas (*Ilex paraguariensis*)	In vivo	When given to obese CB57L/6J mice, Yerba Mate, which is often used as a tea or as an ingredient in prepared food, reduces the differentiation of preadipocytes and the accumulation of lipids in adipocytes, resulting in decreased body weight gain. It also decreases food intake while increasing energy expenditure and basal metabolism.	Yerba Mate has the potential to treat obesity and diabetes, since it enhances glucolipid metabolism.	Kang et al. (2012)
Essential oil of Hedgenettle (*Stachys* spp.)	In vitro	Hedgenettle essential oils have been shown to have antidiabetic and antiobesity properties by inhibiting α‐glucosidase and lipase, respectively.	Essential oil from this herbal tea are significant antiobesity food ingredients due to their bioactive components.	Bahadori et al. (2020)
Moringa seed oil	In vivo	A combination of moringa seed oil extract and lycopene alleviated HFD‐induced hematological and metabolic changes while also lowering leptin and resistin levels. It achieved these results by increasing antioxidant enzymes and decreasing lipid peroxidation, as well as inflammatory cytokines and iNOS protein production.	In HFD‐induced obesity in male Sprauge Dawely rats, *Moringa oleifera* seed oil extract and lycopene show antiobesity potential.	Kilany et al. (2020)
Moringa extract	In vivo	*Moringa oleifera* extract administration to obese rats decreased mRNA expression of leptin and resistin while increasing adiponectin gene expression. This resulted in a decrease in body weight and an improvement in the atherogenic index, coronary artery index, glucose level, and an insulin resistance value.	*Moringa oleifera* has the potential to be beneficial as an antiobesity agent since it works directly on the adipokines of visceral adipose tissue.	Metwally et al. (2017)
Legumes				
Beans (fermented paste)	In vivo	Doenjang administration to C57BL/6J mice given a high‐fat diet decreased body and adipose tissue weight while also decreasing oxidative stress indicators, proinflammatory adipokines, macrophage markers, and fibrosis markers.	Doenjang may be useful in reducing oxidative and systemic inflammation in obesity by inhibiting inflammatory signals in adipose tissue.	Nam et al. (2015)
Meat and meat products				
Sausages	In vitro	The incorporation of lotus seed epicarp into Chinese Cantonese sausage inhibited lipid oxidation while also suppressing in vitro preadipocyte development in 3T3‐L1 cells, resulting in antioxidant benefits.	Lotus seed epicarp has antiobesity properties and may thus be utilized as a healthy addition to Chinese Cantonese sausage.	Qi and Zhou (2013)
Others				
Beverages	In vivo (a nematode)	Beverage prepared with *Cyclocarya paliurus* and *Momordica charantia* decreased oxidative stress damage, fat buildup, and improved lipid profile in *Caenorhabditis elegans*.	This beverage may be useful in the management of oxidative stress and obesity.	Lin et al. (2021)
Yoghurt	Human	Consuming yoghurt fortified with whey protein, inulin, calcium, and vitamin D3, in conjunction with a calorie‐restricted diet decreased body fat mass and waist circumference while enhancing the lipid profile and quantitative insulin sensitivity check index.	Fortified yoghurt may be useful in the treatment of obesity and other metabolic diseases.	Mohammadi‐Sartang et al. (2018)
3D‐printed jelly	In vivo	3D‐printed jelly made from *chenpi*, kiwifruit juice, and pectin was found to lower food intake, liver weight, and fat tissue weight in mice. It also reduced proinflammatory factors (IL‐6 and TNF‐α) in the blood while enhancing antioxidant enzymes.	3D‐printed fruit‐based products may be useful as antiobesity agents.	Peng et al. (2022)
Honey bee propolis	In vitro/in silico	In vitro and in silico Australian honey bee propolis inhibited α‐glucosidase, α‐amylase, and lipase activity.	Honey bee propolis may be useful in the treatment of obesity and diabetes.	Uddin et al. (2022)
Unsaturated alginate oligosaccharides	In vivo	When unsaturated alginate oligosaccharides were employed as food additives, they lowered body weight, serum lipids, free fatty acids, and liver weight. It also raised the phosphorylation of AMP‐activated protein kinase and acetyl‐CoA carboxylase in adipocytes, showing that its antiobesity impact is primarily mediated through AMPK signaling.	Unsaturated alginate polysaccharides have the potential to help cure metabolic illnesses such as fatty liver, obesity, hypertriglyceridemia, and diabetes.	Li et al. (2019)
Starch	In vivo	When fed a high‐fat diet produced obese C57BL/6J mice, a whole grain‐like starch synthesized by co‐gelation of oat β‐glucan and alginate decreased body weight gain, white adipose tissue cell size, and improved lipid profile. It works by suppressing the lipogenic transcription factors SREBP‐1c and fatty acid synthase while increasing the lipid oxidationtranscription factors peroxisome proliferation activated receptor‐α (PPAR‐α) and phosphorylated AMP‐activated protein kinase (p‐AMPK).	As an antiobesity agent, whole grain‐like starch has the potential to be beneficial.	Luo et al. (2018)
Vegetables				
Vegetable juice	In vitro/in vivo	When given to C57BL/6J mice, vegetable juice produced from a mix of *Brassica oleracea*, *Daucus carota*, and *Beta vulgaris* combined with LAB isolated from kimchi decreased weight growth and fat buildup. Its fermentation metabolites were also discovered to prevent the fat formation in human mesenchymal stem cells in vitro.	LAB‐fermented vegetable juice and its metabolites may be useful in the treatment of obesity.	Lee et al. (2023)
Vegetables	Human	Diabetic patients' fecal microbiota was considerably altered by the administration of high‐fiber, polyphenol‐rich, and vegetable protein functional meals, which increased helpful bacteria while decreasing dangerous bacteria. It also helped with glucolipid metabolism.	Long‐term adherence to a high‐fiber, polyphenol‐rich, vegetable protein‐based diet improves intestinal microbiota composition and may offer prospective treatments for improving glycemic control, dyslipidemia, and inflammation.	Medina‐Vera et al. (2019)

## 4 DISCUSSION

Obesity has reached epidemic proportions worldwide, with rising incidence and prevalence in both developed and developing nations (Williams et al., 2013). Despite the disease's global prevalence, many of the drugs in use are untrustworthy and have several unwanted side effects (Mir et al., 2019). Several food crops as well as functional foods have been investigated for antiobesity effects and their potential demonstrated via various modes of action. Many of the food crops and functional food components are widely available, inexpensive, safe, and effective in the treatment of obesity.

Based on the survey, it was found that the bulk of the articles originate from Asia, with Europe and Africa following closely behind. This may be because, due to dietary preferences, genetics, and lifestyle choices, obesity was not often an issue in East Asia prior to the onset of globalization and the nutrition shift. The rising prevalence of obesity has therefore prompted studies on these foods and their characteristics in an effort to learn more and potentially develop strategies to reduce obesity (Mathis et al., 2022). But research on obesity is sparse in Africa because the continent's academics are primarily focused on the existence of other comorbidities and infectious diseases, such as undernutrition (Azeez, 2022).

Since obesity has a multifactorial origin, various therapy components work at different times to alleviate the disease's effects. Various bioactive components or compounds exhibit antiobesity activity via various methods. Certain crops combine more than one mode of action, causing their antiobesity activity to be exerted at multiple sites and resulting in a synergistic impact. Different approaches were employed in the investigations, however this had no bearing on the outcomes or conclusions because the mechanisms of actions could still be determined. Some of the processes discovered in this review include the regulation of gut microbiota, as proven by Gong et al. (2019)), and the inhibition of adipogenesis (Kang et al., 2016). Whole or fermented cereals alter the gut microbiota composition and glucolipid metabolism. In addition, anthocyanin in pigmented cereals, some polyphenol compounds, and high dietary fiber contents of cereal grains are also responsible for their antiobesity effects. Some plants combine two or more methods; Okouchi et al. (2019) hypothesized that simultaneous consumption of the edible mushroom *Euglena gracilis* with vegetables inhibits genes implicated in fatty acid metabolism while also modifying gut microbiota. Additionally, several plants have an antiobesity effect by blocking glucolipid metabolism enzymes such as α‐glucosidase, α‐amylase, and lipase (Fabroni et al., 2016). Because diverse groups of food crops or plants such as fruits contain distinct bioactive (phenolics, flavonoids, anthocyanins, polysaccharides, and seed proteins) chemicals that are responsible for their actions, these different dietary classes can function in different ways.

Gut microbiota is an essential player in the control of host energy balance and intestinal permeability, and its composition has been linked to the development of numerous metabolic illnesses and physical well‐being. The composition of the diet is one of the most important elements influencing gut microbiota, and various food crops, food components, or bioactive compounds with prebiotic qualities can aid in the treatment of specific disorders by modifying the composition of gut microbes (Jiang et al., 2021). Foods rich in dietary fiber, such as grains (barley) as reported by Gong et al. (2019)), unripe plantain flour (Fu et al., 2022), and edible mushrooms such as *E. gracilis* (Okouchi et al., 2019), might fuel the production of short‐chain fatty acids (SCFAs) and increase the quantity of beneficial gut microbiota (Jiang et al., 2021). The gut microbiota aids in the extraction of surplus energy from the meal. The gut microbiome also promotes the expression of critical transcriptional factors that promote lipogenesis in the liver and lipoprotein lipase (LPL) activity in adipocytes to store triglycerides (TG). There needs to be a balance in the composition of the gut microbiota as previous reports showed that obese patients had fewer Bacteroidetes and more Firmicutes in their gut, with the proportion of Bacteroidetes rising with the commencement of a reduced calorie diet (Dahiya et al., 2017). Laminarin (Nguyen et al., 2016), blueberry polyphenols (Jiao et al., 2019), and flaxseed polysaccharides (Yang et al., 2020) are some biomolecules or bioactive components present in food crops that have been shown (in the publications evaluated in this study) to have antiobesity actions through modifying gut microbiota. Blueberry polyphenols, laminarin, and flaxseed polysaccharides, as well as other food ingredients like dietary fiber, function as prebiotics, increasing the composition of Bifidobacterium and Proteobacteria (beneficial bacteria; Jiao et al., 2019; Nguyen et al., 2016; Yang et al., 2020). A high‐calorie diet has been shown to increase the number of nonbeneficial bacteria Firmicutes in the gut while decreasing the population of helpful bacteria Bacteroidetes. However, the energy harvesting capacity of the gut is primarily due to the activities of carbohydrate‐active enzymes (CAzymes) produced by Bacteriodetes species; thus, a high‐fat diet leads to obesity because energy intake exceeds expenditure, and a decrease in the population of Bacteriodetes means that the energy harvesting role of the CAzymes they produced is not achieved (Flint et al., 2012).

Since excessive fat and carbohydrate consumption causes triglyceride accumulation in the liver and adipose tissues, increasing the risk of developing obesity, hyperlipidemia, and other complications, inhibiting enzymes such as pancreatic lipase, involved in glucolipid metabolism is another mechanism by which obesity can be managed or prevented (Jiang et al., 2021). Pancreatic lipase is in charge of converting triglycerides into mono‐ and diglycerides, as well as tiny fatty acids that are absorbed by the body. As a result, inhibiting it can diminish fat digestion and assimilation and absorption, avoiding weight gain (Gooda Sahib et al., 2012). Another enzyme is lipoprotein lipase, it hydrolyzes triglycerides in the blood to release free fatty acids and so enhances triglyceride storage in adipose tissues, and its inhibition lowers the accumulation and assimilation of free fatty acids and hence controls obesity (Gooda Sahib et al., 2012). Other enzymes, such as α‐amylase and α‐glucosidase, which aid in carbohydrate digestion and metabolism, when inhibited, diminishes postprandial hyperglycemia and hence delay the conversion of glucose in adipose tissue to triglycerides (Gooda Sahib et al., 2012). Some documents evaluated in this review precisely looked into this mechanism; anthocyanins in vegetables, fruits, and legumes inhibit the activity of pancreatic lipase (Fabroni et al., 2016), *Daucus carota* exerts its antiobesity effect via inhibition of pancreatic lipase (Marrelli et al., 2020), and Mediterranean dietary plants exert their antiobesity effect by inhibiting pancreatic lipase and α‐amylase (Mariangela Marrelli et al., 2014). Phenolics are one of the bioactive compounds that have been found to function by this method (Ranilla et al., 2019). Phenolics including quercetin, catechin, and luteolin block the digestive enzymes α‐amylase, α‐glucosidase, and lipase (Song et al., 2019). Phenolics in *Taraxicum officinale* (myricetin, isomangeferin, and kaempferol) inhibit pancreatic lipase (Aabideen et al., 2020). Teas modified with immobilized tannase to change their phenolic content had improved inhibitory action against the digestive enzymes α‐amylase, α‐glucosidase, and lipase (Roberto et al., 2016). Moreover, Coronado‐Cáceres et al. (2020)) found that cocoa seed proteins have an antiobesity impact by reducing the activity of pancreatic lipase.

Improving energy expenditure and thermogenesis is another mechanism used in the treatment of obesity. With mitochondria accounting for the majority of energy consumption, increased uncoupling of mitochondrial oxidative phosphorylation may boost heat production (Jiang et al., 2021). White adipose tissue (WAT) and brown adipose tissue (BAT) are the two forms of adipose tissue (BAT). BAT has a large number of mitochondria, which are vital in thermogenesis mediated by uncoupling protein 1 (UCP1) and activation of AMP‐activated protein kinase (AMPK), which helps to convert WAT into BAT and upregulate thermogenic genes (Hu et al., 2020). AMPK activation then activates peroxisome proliferator‐activated receptor‐γ coactivator 1α (PGC1a), which speeds lipid catabolism and increases mitochondria and UCP1 synthesis, enhancing energy expenditure and thermogenesis (Hu et al., 2020). As a result, various plants or food crops, as well as bioactive substances, have been found to produce browning of adipose tissues or to upregulate genes linked with thermogenesis, which improves energy expenditure and reduces obesity. The phenolic acids *trans*‐cinnamic acid and ellagic acid, as well as other phenolic compounds such as resveratrol, anthocyanins, and catechins, have been found to function via this mechanism (Hu et al., 2020). Kang et al. (2016)) examined this mechanism on the antiadipogenic activity of the edible mushroom *Pleurotus eryngii*, and Malarvizhi et al. (2021) reported that *Macrotyloma uniflorum* modulates adipokines and peroxisome proliferator activator receptors (PPARs). Gu et al. (2021) described how fermented barley induces WAT browning, and Wang et al. (2019)) investigated ginger and how it affects the thermogenesis and browning of white adipose tissues. *Trans*‐cinnamic acid has also been studied for its capacity to treat obesity via thermogenesis and adipose browning (Z. Wang et al., 2020). Another bioactive chemical that has been linked to the browning of white adipose tissues and thermogenesis is fucoxanthin (Gille et al., 2019). Li et al. (2019) also discovered that unsaturated alginate polysaccharides mediate AMPK signaling, lowering adipocyte weight.

Furthermore, excess dietary energy is typically stored as fat in adipose tissues, resulting in adipose tissue hypertrophy and hyperlipidemia. The prevalence of obesity has been linked to adipose tissue hypertrophy (Gooda Sahib et al., 2012). When the body requires energy, this stored fat is hydrolyzed into fatty acids, which are then oxidized to supply energy (Jiang et al., 2021). Thus, modulating the processes of lipogenesis and lipolysis is a very promising strategy for combating obesity and related disorders. Okouchi et al. (2019) reported that the combination of modulation of gut microbiota and suppression of lipogenesis are mechanisms employed by the simultaneous intake of *Euglena eryngii* and vegetables in combating obesity. Another study (Na et al., 2019) found that a fermented extract of *Chaga‐cheonggukjang* suppressed lipogenesis as a mechanism for combating obesity. Mulberry leaf polyphenols have also been shown to suppress the expression of genes involved in lipogenesis (Chang et al., 2016). Several studies (Ali et al., 2015; Diez‐Echave et al., 2020; Ji et al., 2021; Lee et al., 2022; Tamaru et al., 2013) also investigated this mechanism. Some of the enzymes involved in fatty acid synthesis are fatty acid synthase and stearoyl‐CoA desaturase (SCD), and the genes responsible for these enzymes are activated by specific transcription factors such as sterol regulatory element binding protein1c (SREBP1c), carbohydrate‐responsive element‐binding protein (ChREBP), and upstream stimulating factors (UCFs; Wang et al., 2015). A malfunction of these factors inhibits the expression of genes involved in the synthesis of fatty acids and cholesterol, reducing obesity and other obesity‐related complications such as hyperlipidemia, hypertriglyceridemia, hypercholesterolemia, and insulin resistance (Wang et al., 2015). As a result, some compounds target these specific transcription factors to combat obesity. Inducing fatty acid oxidation to reduce lipid storage is another strategy used to combat obesity; thus, food crops or bioactive molecules that modulate fatty acid oxidation regulators such as PPAR‐γ, carnitine palmitoyl transferase (CPT1), and sirtuin 3 (SIRT3) aid in the fight against obesity (Jiang et al., 2021). Polyphenols, polysaccharides, and plant peptides are examples of bioactive molecules that have been reported to have this effect (Xie et al., 2018). Green tea is one food crop or plant that has been reported to use this mechanism. Green tea epigallocatechin gallate (EGCG) has been shown in several studies to inhibit proliferation and differentiation in primary human visceral preadipocytes. Thus, EGCG's ability to promote weight loss may be due in part to its ability to suppress the number of adipocytes as well as triacylglycerol uptake (Gooda Sahib et al., 2012).

Prebiotics and probiotics are well‐known functional foods for their beneficial effects on gastrointestinal health. Probiotics are live microorganisms that, when administered in sufficient amounts, benefit the host's health, whereas prebiotics are indigestible food ingredients that are fermented by gut microbiota, serve as a substrate selectively utilized by host microbiota, and confer health benefits (Gibson et al., 2017). Prebiotics' antiobesogenic effects include improvements in glucose and lipid metabolism, as well as glycemic control. Probiotics have a wide range of effects on the host, including antagonistic effects on various microbiota and competitive adherence to the mucosa and epithelium (antimicrobial activity), increased mucus production and barrier integrity (enhanced barrier function), and immunomodulation (Hijova, 2021). Fermented products such as yoghurt are examples of functional foods enriched with probiotic microbiota that improve digestive health. Their combination (probiotics and prebiotics), known as “synbiotics,” may serve to enhance the beneficial probiotic effects even further (Hijova, 2021). Obese subjects who consumed probiotic‐enriched yoghurt had a lower waist circumference and body percentage, as well as lower triglyceride levels and increased insulin sensitivity when compared to those who consumed plain yoghurt (Mohammadi‐Sartang et al., 2018). *Doenjang*, the Korean fermented soybean paste is another functional food in this review with antiobesogenic activity due to its prebiotic and probiotic effects (Nam et al., 2015).

Teas are unfermented beverages high in polyphenolic compounds that are consumed globally. Tea polyphenols have been shown to inhibit pancreatic lipase and thus influence lipid metabolism (Yuda et al., 2012). Tea contains caffeine, which has been shown to have thermogenic effects and stimulate fat oxidation. Caffeine and polyphenols in tea work together to promote fat oxidation (Sunkara & Verghese, 2014). Green tea is thought to increase energy expenditure by inhibiting the enzyme catechol O‐methyltransferase (COMT), which degrades norepinephrine, inhibiting the enzyme phosphodiesterase, which degrades intracellular cAMP, antagonizing the inhibitory effects of adenosine on lipolysis, increasing the expression of uncoupling proteins, and activating PPARs (Sunkara & Verghese, 2014). Several studies reported the antiobesogenic effect of mixed tea, green tea modified with immobilized tannase, and Fuzhuan brick tea (Li et al., 2013; Roberto et al., 2016; Tamaru et al., 2013). These teas also suppress lipogenesis while increasing thermogenesis in adipose tissues. Another functional food reported in this study is kiwifruit jelly, which was developed using 3D printing technology with kiwifruit juice, *chenpi*, and pectin. This snack inhibited adipogenesis by improving metabolic homeostasis and increasing satiety (Peng et al., 2022). Several of these food groups are commonplaces across a wide range of cultures and geographical areas and can be added to diets, used as fortifiers or supplements, or included as a core component of dietary patterns. With the various food classes shown in these research, there are numerous alternatives available to those seeking dietary interventions for the management of obesity, and regardless of personal preference, there is something to suit everyone. In certain cases, lifestyle interventions such as diet and exercise can effectively manage the condition depending on its severity. However, in cases where the disease is chronic, medical interventions such as bariatric surgery or the use of medications like semaglutide can be combined with lifestyle interventions to achieve the best possible outcome (Busetto et al., 2023).

## 5 CONCLUSION

Obesity develops through several complex pathways, and most conventional drugs target only one of these pathways and must be used in conjunction with other drugs to be effective in the management of the disease. However, these conventional drugs have a variety of side effects, so plant materials and natural products have recently been targeted in the treatment of obesity. This review article identifies several food crops and functional foods have an antiobesogenic effect by targeting more than one pathway involved in the etiology of the disease, making them very effective and safe while also providing nutritional benefits. Glucolipid metabolism is the most dominant pathway explored in this review, more food crops and developed functional food may be explored using this pathway and others, to promote their consumption as a means of effectively managing this global menace. It has been shown that a combination of genetic, epigenetic, and environmental factors contribute to obesity. Since genetic factors are uncontrollable, dietary habits are one of the major environmental factors that can be changed to address obesity. Additionally, other factors like socioeconomic status have an impact on dietary choices; therefore, for optimal outcomes, all of these factors must be taken into account and controlled. However, many of the studies had limited subjects and sometimes in vitro studies are difficult to extrapolate to human studies. Further research studied should be geared towards the exploration of underutilized and indigenous food crops, their innovative processing and applications, and evaluating their safety and efficacy in eradicating obesity. This will strengthen local research and development capabilities in Africa, and assist in achieving public health goals.

## AUTHOR CONTRIBUTIONS

**Beatrice Mofoluwaso Oladimeji:** Conceptualization (equal); data curation (lead); formal analysis (lead); methodology (equal); software (lead); writing – original draft (lead); writing – review and editing (equal). **Oluwafemi Adebo:** Conceptualization (equal); funding acquisition (lead); methodology (equal); supervision (lead); writing – review and editing (equal).

## CONFLICT OF INTEREST STATEMENT

The authors have no conflict of interest to declare.

## Data Availability

Data sharing not applicable to this article as no datasets were generated or analyzed during the current study.
